# Synthesis and Photocatalytic Properties of CuO-CuS Core-Shell Nanowires

**DOI:** 10.3390/ma12071106

**Published:** 2019-04-03

**Authors:** Yuan-Tse Kao, Shu-Meng Yang, Kuo-Chang Lu

**Affiliations:** 1 Department of Materials Science and Engineering, National Cheng Kung University, Tainan 701, Taiwan; jinnii10377@yahoo.com.tw (Y.-T.K.); young263263@gmail.com (S.-M.Y.); 2 Center for Micro/Nano Science and Technology, National Cheng Kung University, Tainan 701, Taiwan

**Keywords:** copper oxide, copper sulfide, thermal oxidation, nanowires, transmission electron microscopy, photocatalyst

## Abstract

In this study, an efficient method to synthesize CuO-CuS core-shell nanowires by two-step annealing process was reported. CuO nanowires were prepared on copper foil via thermal oxidation in a three-zone horizontal tube furnace. To obtain larger surface area for photocatalytic applications, we varied four processing parameters, finding that growth at 550 °C for 3 h with 16 °C/min of the ramping rate under air condition led to CuO nanowires of appropriate aspect ratio and number density. The second step, sulfurization process, was conducted to synthesize CuO-CuS core-shell nanowires by annealing with sulfur powder at 250 °C for 30 min under lower pressure. High-resolution transmission electron microscopy studies show that a 10 nm thick CuS shell formed and the growth mechanism of the nanowire heterostructure has been proposed. With BET, the surface area was measured to be 135.24 m^2^·g^−1^. The photocatalytic properties were evaluated by the degradation of methylene blue (MB) under visible light irradiation. As we compared CuO-CuS core-shell nanowires with CuO nanowires, the 4-hour degradation rate was enhanced from 67% to 89%. This could be attributed to more effective separation of photoinduced electron and hole pairs in the CuO-CuS heterostructure. The results demonstrated CuO-CuS core-shell nanowires as a promising photocatalyst for dye degradation in polluted water.

## 1. Introduction

In recent years, organic pollutants released from industries have caused major environmental damages; thus, various contaminant treatments are being researched, including absorption [1,2], electrolysis [3], chemical flocculation [4], and photocatalytic degradation [5,6,7,8,9,10,11,12]. Among these treatment techniques, photocatalytic degradation is a low-cost, environmentally friendly, and sustainable method for the removal of organic pollutants. A photocatalytic reaction on a semiconductor includes at least five main steps [13], namely, light absorption by the semiconductor, formation of photogenerated electron-hole pairs, migration, and recombination of the photogenerated electron-hole pairs, adsorption of reactants, and desorption of products and occurrence of redox reactions on the semiconductor surface. However, the low efficiency of carrier separation resulting in rapid recombination limits overall efficiencies for the applications in both contaminant removal and renewable energies. To modify the efficiency of carrier separation, introducing heterojunctions in photocatalysts is the most promising strategy by transferring a charge carrier from one semiconductor to another in close contact. However, the most popular photocatalyst, TiO_2_, only works under UV light, which is less than 5% of solar energy on the Earth’s surface; thus, the photocatalytic applications of TiO_2_ are limited. Recently, great efforts have been dedicated to developing visible light-driven photocatalysts to more effectively utilize the solar energy and apply in indoor environment.

The morphology of materials is critical in terms of photocatalytic performance. 1-D nanostructures, such as nanowires, nanorods, and nanobelts, not only have attractive properties, but also have a large surface area, which may enhance photocatalytic efficiency. Among nanowires, transition metal silicides possess low resistivity, high melting temperature, and good stability [14,15,16,17]. As transition metal oxides, nanoscale Cupric oxide (CuO) is being utilized in various applications, including photocatalysts [6,7,8,9,10,11,12], gas sensors [18,19,20,21,22,23], resistive random access memory (RRAM) [24,25,26,27], solar cells [28], and lithium-ion batteries [29,30,31]. The solution-phase method has been employed to synthesize various 2-D or 3-D morphologies of CuO, such as nanospheres [6], nanoflowers [7], nanosheets [8], and straw-sheaf-like nanostructures [9]. Moreover, it can allow large-scale production without much cost. For 1-D CuO nanostructures, the most popular growing method is thermal oxidation due to its high yield and technical simplicity. More importantly, CuO photocatalysts can be driven by visible light because of its p-type semiconductor characteristics with a band gap of around 1.4 eV [32]. Cupric sulfide (CuS) is also a p-type semiconductor with a band gap of 2.08 eV [33,34]. Therefore, CuS is an outstanding photocatalyst owing to the absorption of visible light in the solar spectrum. Various morphologies [33,34,35,36] for the photocatalytic application have been investigated.

In this work, CuO nanowires and CuO-CuS core-shell nanowires were synthesized through thermal oxidation and two-step annealing. Photocatalytic degradation of methylene blue was used to evaluate the photocatalytic properties of CuO nanowires and CuO-CuS core-shell nanowires.

## 2. Materials and Methods

Commercial copper foils from Alfa Aesar with purity of 99.9% and thickness of 0.127 mm were used as the starting material. They were cut into 4 mm × 5 mm pieces, and cleaned in an ultrasonic bath for 10 min with acetone and isopropyl alcohol, respectively; then, they were rinsed with deionized water and dried with a nitrogen gun. The cleaned foils were placed in an alumina boat and under an ambient condition in a three-zone tube furnace to grow CuO nanowires. To synthesize CuO-CuS core-shell nanowires, the second-step annealing process was conducted. Sulfur powder from Sigma Aldrich with purity of 99.98% was used as the precursor to react with as-grown CuO nanowires in the three-zone furnace. The temperature was set at 250 °C with a ramping rate of 7 °C per minute, and the chamber was pumped to 0.05 torr without carrier gas. 

The morphology of CuO nanowires was observed with a Hitachi SU8000 field emission scanning electron microscope (FE-SEM, Tokyo, Japan). The grazing incidence X-ray diffraction (GIXRD) patterns were obtained by an X-ray diffractometer (Bruker D8 DISCOVER with GADDS, Cu K_α_ = 1.5406 Å, Billerica, MA, USA) for crystal structure analysis. High-resolution transmission electron microscope (HR-TEM) images from JEOL JEM-2100F (Tokyo, Japan) equipped with Cs corrector were used to identify the core-shell structure. Scanning transmission electron microscope-energy dispersive spectrometry (STEM-EDS) line scanning was conducted with Philips Tecnai F20 G2 FEI-TEM (Amsterdam, The Netherland). The surface area and pore size were measured by BEL BELSORP mini (Kyoto, Japan).

The photocatalytic properties of the samples were evaluated by the degradation of methylene blue (MB) under two white light LED flashlights (200 lm) irradiation in a black box. A 4 mL of 10 mg/L aqueous MB solution was taken in a 4 mL vial. We took 0.2 mL of sample to a new sample vial per hour, and then added 3 mL of deionized water for UV/Vis spectrometer (PerkinElmer LAMBDA 950, Waltham, MA, US) measurement. The experiment was conducted for a total of 4 h.

## 3. Results and Discussion

### 3.1. Morphologies of CuO Nanowires

**Figure 1:**
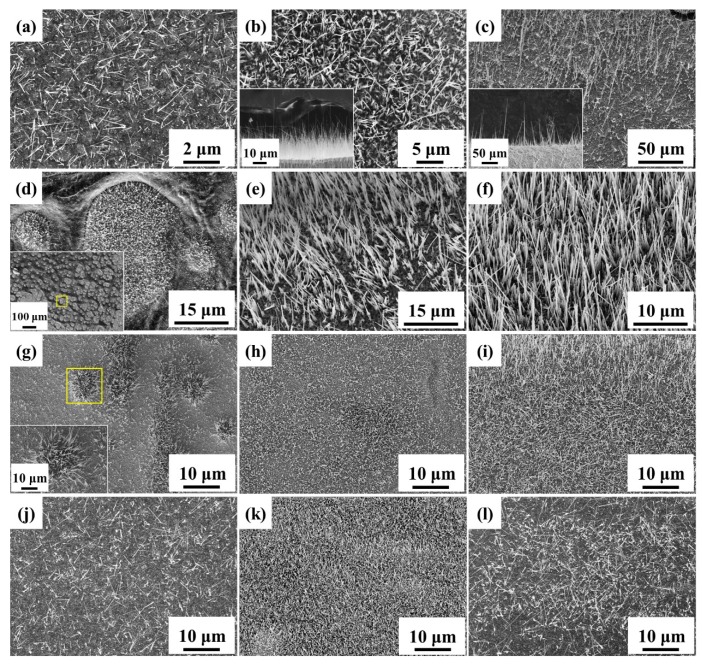
FE-SEM images of CuO nanowires grown at different temperatures (**a**) 350 °C, (**b**) 550 °C, (**c**) 800 °C, for different oxidation time (**d**) 0 h, (**e**) 1 h, (**f**) 2 h, with different ramping rates (**g**) extremely fast, (**h**) 8 °C/min, (**i**) 4 °C/min, and under different oxygen concentrations (**j**) 10%, (**k**) 50%, (**l**) 100%.

Surface area is one of the critical factors for photocatalytic properties; thereby, both high aspect ratio and number density of nanowires are significant to improve the photodegradation rate. In Figure 1, the four processing parameters, annealing temperature, oxidation time, heating rate as well as oxygen concentration were investigated in terms of their influence on the morphology. Unmentioned parameters were fixed at 550 °C of annealing temperature, 3 h of oxidation time, 16 °C/min of ramping rate, and 20% of oxygen concentration (ambient condition). Figure 1a–c shows that the appropriate temperature for growing CuO nanowires was around 550 °C. The growth of CuO nanowires has been previously explained by stress-driven mechanism [37,38,39,40,41]. In the compression region, Cu ions would diffuse along grain boundaries, leading to CuO nanowire growth on CuO grains. At lower temperatures, CuO nanowires grew slowly owing to the low mobility of Cu ions and insufficient stress generation to drive the grain boundary diffusion. However, the interface strain could be released rapidly by lattice diffusion at higher temperatures. As a result, CuO tended to grow uniformly and form flatter morphologies with fewer nanowires. Figure 1d–f show the growth of nanowires from none to many; the length of CuO nanowires followed the parabolic growth law, x = (2kt)^1/2^ [38], where x is the length of CuO nanowires, k is the rate constant, and t is the growth time. Figure 1 g–i are for the effect of ramping rate. The extremely fast ramping rate was conducted by putting the foil into the 550 °C furnace instead of heating from room temperature. Some urchin-like hills with long nanowires were observed due to the uneven relaxation of large stress. With the decline of heating rate, the morphology became uniformly distributed nanowires. Additionally, Figure 1j–l shows the results under different oxygen concentrations through varying the ratio of nitrogen and oxygen gas in the chamber and keeping the total pressure at 1 atm. It was found that the medium oxygen concentration contributed to the better morphology for the suitable surface oxidation rate. 

After comparing the four processing parameters, we fixed the parameters at 550 °C for 3 h with 16 °C/min of ramping rate and 20% of oxygen concentration for subsequent experiments.

### 3.2. Synthesis and Characterization of CuO-CuS Core-Shell Nanowires

**Figure 2:**
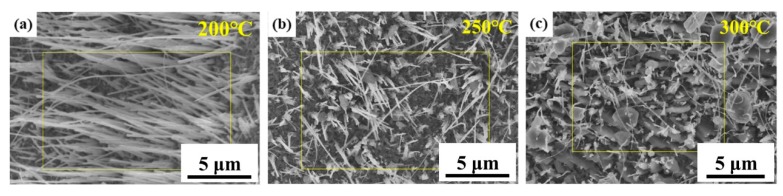
FE-SEM images of CuO nanowires under sulfurization process at different temperatures (**a**) 200 °C, (**b**) 250 °C, (**c**) 300 °C.

Sulfurization process was then conducted for as-grown CuO nanowires. After the second annealing procedure, all the sulfur powder was evaporated and pumped out completely. The morphology under FE-SEM is shown in Figure 2. At 200 °C, CuO nanowires barely reacted with sulfur vapor since the temperature was not high enough to overcome the activation energy. At 250 °C, the surface of CuO nanowires became rough and the sulfur signal could be detected in EDS of SEM. At 300 °C, some huge particles were found, and the structure of the nanowires was destroyed because of the high temperature; the rapid reaction between CuO and sulfur vapor and the fast diffusion of Cu^2+^ in CuO led to large consumption of CuO.

**Figure 3:**
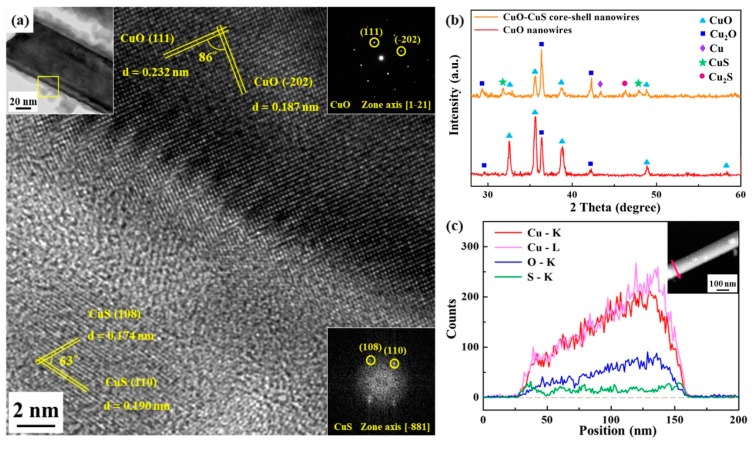
(**a**) TEM and HR-TEM images, (**b**) XRD analysis, as well as (**c**) line scanning profile and high angle annular dark field (HAADF) image of CuO-CuS core-shell nanowire(s).

Further characterization for CuO nanowires was conducted after 250 °C sulfurization process. Figure 3b shows the X-ray diffraction (XRD) pattern, where both CuO and Cu_2_O show major peaks. The inset of Figure 2b reveals a hierarchical structure. The literatures [39,40,41] show the same results that the bottom layer is Cu_2_O and the top layer are CuO nanowires and thin-film, respectively. After the sulfurization process, the relative intensity of CuO peaks comparing with Cu_2_O is weaker due to the consumption of CuO for the formation of copper sulfide. The characteristic peaks at 2θ = 31.78° and 47.94° correspond to CuS (103) and (110), respectively. Also, another characteristic peak at 2θ = 46.10° was detected, resulting from Cu_2_S (220). The reappearance of Cu (111) at 2θ = 43.30° was attributed to the consumption of the CuO layer for reacting with sulfur vapor. Cu_2_S was absent in the TEM analysis of CuO-CuS core-shell nanowire; it might have been formed at the root of CuO nanowires with sulfur vapors of insufficient concentration. Here, we propose the main chemical reactions as follows:

2 CuO_(s)_ + 3 S_(g)_ → 2 CuS_(s)_ + SO_2(g)_, (i)

when sulfur is limited:

2 CuO_(s)_ + 2 S_(g)_ → Cu_2_S_(s)_ + SO_2(g)_, (ii)

Figure 3a reveals the TEM analysis for the nanowire heterostructure, including the low magnification image, corresponding selected area electron diffraction (SAED) pattern of the core part, fast Fourier transform (FFT) pattern of the shell part and high-resolution transmission electron microscopy (HR-TEM) image. A core-shell nanowire of 100 nm in diameter with about 10 nm thick shell could be observed in the low magnification image. Based on the EDS analysis, the atomic ratio of Cu to O for the core part is nearly 1:1, while that of Cu to S for the shell part is also nearly 1:1. From HR-TEM analysis, we can index (111) and (-202) with lattice spacings of 0.232 nm and 0.187 nm, respectively, confirming the core to be monoclinic CuO; for the shell, two lattice planes of (110) and (108) with lattice spacings of 0.190 nm and 0.174 nm, respectively, were indexed and the shell has been identified to be hexagonal CuS. Figure 3c shows the EDS line scanning operated under STEM mode; the inset is high angle annular dark field (HAADF) image, looking lighter in color at the right half of the nanowire since the nanowire is not a perfect cylinder, being thicker at that area. Also, a ~10 nm thick shell in a nanowire can be seen in the line scanning profile. Additionally, we conducted the nitrogen adsorption-desorption measurements as shown in Figure S1 and Table S1. The surface area and pore size of CuO-CuS nanowires are 135.24 m^2^/g and 1.7 nm, respectively.

**Figure 4:**
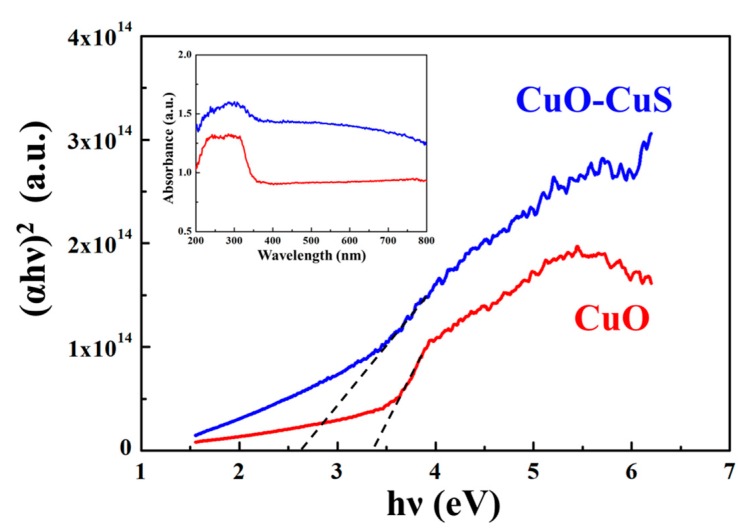
Tauc plot of CuO-CuS and CuO nanowires. The inset is UV-Vis diffuse reflectance spectra (DRS).

Figure 4 shows the Tauc plot and UV-Vis diffuse reflectance spectra (DRS) for CuO-CuS and CuO nanowires. The significant absorptions of the CuO-CuS and CuO nanowires were at 310 and 240 to 320 nm, respectively. According to the equation, αhv = C(hv − Eg)^1/2^, and the direct transition, a plot of (αhv)^2^ vs. hv contributes to a curve and the band gap energy of the CuO-CuS and CuO nanowires can be calculated by extrapolating the linear portion of the curve to the hv axis, where α is the absorption coefficient, hv is the photon energy, C is a constant, and Eg is the band gap energy. The band gap energies of the CuO-CuS and CuO nanowires were calculated to be 2.6 eV and 3.3 eV, respectively. The band gap energy of CuO nanowires is larger than that of bulk CuO, which may be due to the blue shift of the direct band gap, affected by the morphologies of the crystal and the quantum size effect. The band gap energy of CuO-CuS nanowires is smaller than that of CuO nanowires and within the visible light range; thus, we can choose the visible light as the source in photocatalytic reactions instead of inaccessible UV light of higher energy.

### 3.3. Photocatalytic Properties

**Figure 5:**
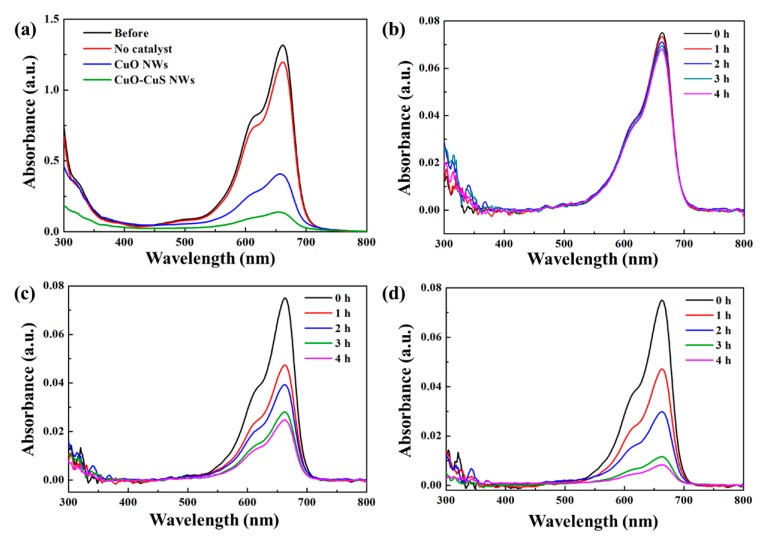
(**a**) UV-Vis absorbance spectra of MB aqueous solutions before and after 4 h visible light irradiation as well as UV-Vis absorbance spectra on MB degradation at different durations for (**b**) no catalyst (**c**) CuO nanowires (**d**) CuO-CuS core-shell nanowires.

**Figure 6:**
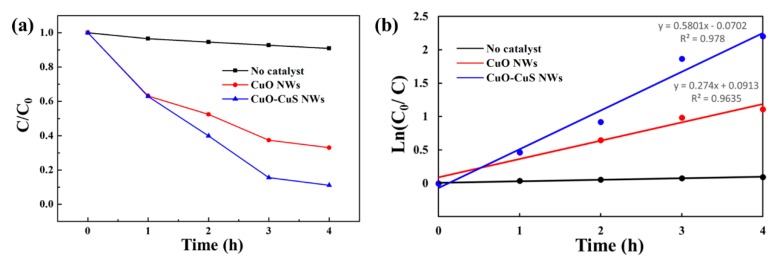
(**a**) Relative concentration (C/C_0_) of MB versus visible light irradiation time, and (**b**) ln(C_0_/C) versus time for kinetic analysis of MB degradation.

The photocatalytic properties of CuO nanowires and CuO-CuS core-shell nanowires were studied for the efficient degradation of methylene blue dye. The photocatalytic degradation of methylene blue was monitored under visible light irradiation and the measurement was conducted with a UV–vis spectrophotometer. The absorbance values of the characteristic absorption peak at 662 nm were used to calculate the dye degradation rate. According to the Beer–Lambert’s law, the concentration of MB is linearly proportional to the intensity of the absorption peak at 662 nm; thus, the decomposition rate of MB can be calculated using the following formula:

(1) $$\mathrm{Degradation}\;\mathrm{rate}\;=\;\left(1-\;\frac{C}{C_{0}}\right)\;\times \;100\;\%,$$

where C_0_ is the initial concentration before degradation and C is the concentration after time. Figure 5a shows the UV-Vis absorbance spectra for aqueous solutions of MB before and after 4h visible light irradiation for no catalyst, CuO nanowires, and CuO-CuS core-shell nanowires. Figure 5b–d are UV-Vis absorbance spectra on MB aqueous solution at different durations for the three samples, respectively. The relative concentration of the three samples is shown in Figure 6a. A blank experiment was conducted with the absence of the catalyst under the visible light irradiation and the experiment shows that only about 9% of dye was degraded. The degradation rate of MB for CuO nanowires was 37%, 47%, 63% and 67% after 1, 2, 3 and 4 h, respectively. For CuO-CuS core-shell nanowires, the degradation of MB dye solution was found to be 37%, 60%, 84% and 89% after 1, 2, 3 and 4 h, respectively, which is an apparent improvement as compared with as-grown CuO nanowires. The degradation of MB followed pseudo-first-order kinetic model with a rate constant of 0.274 h^−1^ for CuO nanowires and that of 0.580 h^−1^ for CuO-CuS core-shell nanowires.

**Figure 7:**
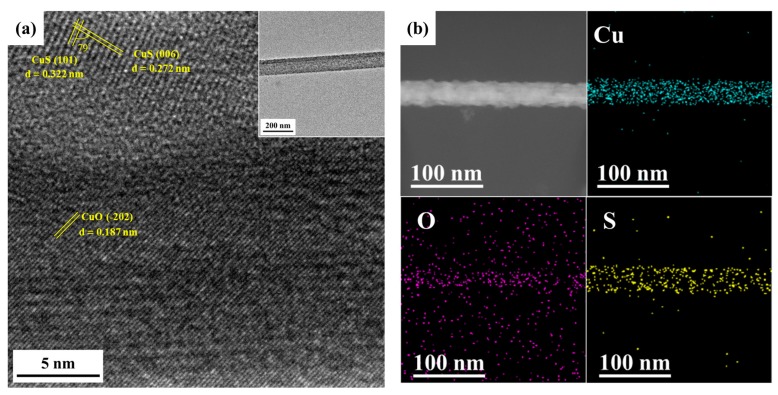
(**a**) TEM analysis and (**b**) EDS mapping of the CuO-CuS nanowire after 4 cycles of photocatalytic reactions

The stability of the CuO-CuS nanowires has been investigated by structural and surface analysis after the photocatalytic reaction. TEM images of the CuO-CuS nanowires taken after four photocatalytic reaction cycles are shown in Figure 7. It can be seen that the crystal structures of CuO and CuS did not change and the core-shell structure was maintained well. Additionally, no corrosion of nanowires was found. Also, Figure S2 shows the results of the four photocatalytic reaction cycles. The absorbances of MB at 662 nm did not vary apparently in every cycle. The absorbance decayed just 3% from the first to the second time; namely, the degradation of MB after 4 h of reaction decayed from 89% to 86% and then kept stable. These results demonstrate the excellent photocatalytic stability of the CuO-CuS core-shell nanowires in harsh photocatalytic reactions. 

The photocatalytic reaction is explained here. When photocatalysts were irradiated by visible light with photon energy larger or equal to the band gap of photocatalysts, electrons in the valence band could be excited to the conduction band; as a result, the photo-induced electron-hole pairs were generated. The photogenerated electron-hole pairs would recombine or react with oxygen and water to form superoxide radicals and hydroxyl radicals, which are extremely strong oxidants for organic compounds. The increase in degradation rate could be attributed to the staggered gap [13] between CuO and CuS.

**Figure 8:**
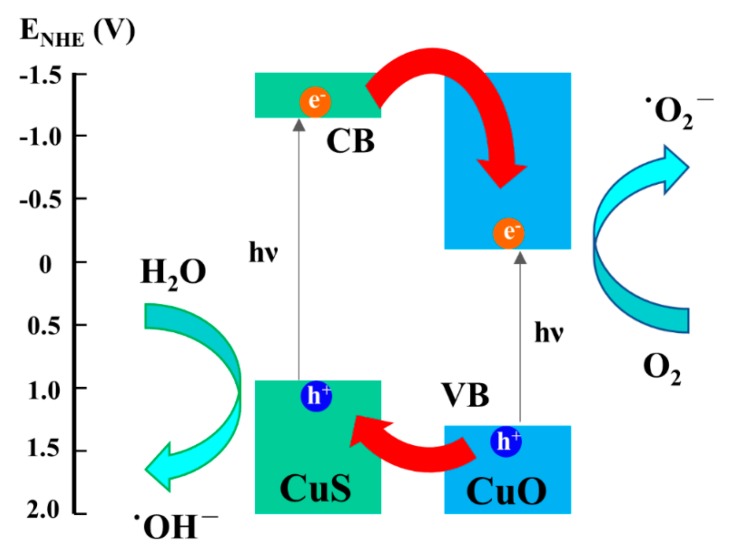
Schematic illustration of photocatalytic mechanism for the CuO-CuS nanowire heterostructure.

As schematized in Figure 8, the conduction band of CuS is more positive in potential than CuO; thus, the photogenerated electrons will transfer from CuS to CuO. On the other hand, the valence band of CuO is more negative in potential than CuS; thereby, the photogenerated holes will migrate from CuO to CuS. The spatial segregation of electron-hole pairs could inhibit the recombination of photogenerated electron-hole pairs, leading to the promotion of photocatalytic efficiency. The spatial segregation of electron-hole pairs could inhibit the recombination of photogenerated electron-hole pairs, leading to the promotion of photocatalytic efficiency.

## 4. Conclusions

In summary, we investigated the morphology of CuO nanowires via thermal oxidation to obtain a larger surface area for photocatalytic applications. Through the sulfurization process with sulfur powder under low pressure, CuO-CuS core-shell nanowires were successfully synthesized. The structure of the core-shell was characterized by high-resolution transmission electron microscopy. The surface area of the nanowire was measured by BET. The CuO-CuS core-shell nanowires, the band gap energy of which was calculated by DRS to be within the visible wavelength range, could facilitate the separation of the photogenerated electrons and holes, and enhance photocatalytic activity under visible light irradiation. TEM analysis reveals that the core-shell structure of CuO-CuS was maintained well after four cycles of photocatalytic reactions. This work provides an efficient and economical method for eliminating pollution and environmental protection.

## Supplementary Materials

The following are available online at https://www.mdpi.com/1996-1944/12/7/1106/s1, Figure S1: (a) Nitrogen adsorption-desorption isotherms and (b) corresponding pore size distribution curve of the CuO-CuS nanowires, Table S1: BET surface area and pore size of the CuO-CuS nanowires, Figure S2: UV-Vis absorbance spectra for cycling degradation of MB aqueous solutions by CuO-CuS nanowires.
