# Supplementary material for: Synthesis and Photocatalytic Properties of CuO-CuS Core-Shell Nanowires
Source: Materials (Basel). 2019 Apr 3;12(7):1106. doi: 10.3390/ma12071106 (PMC6479540; doi:10.3390/ma12071106)
Supplement: Supplementary file 1 [file materials-12-01106-s001.pdf]

Supporting Information

Synthesis and Photocatalytic Properties of CuO-CuS Core-Shell Nanowires

Yuan-Tse Kao [1], Shu-Meng Yang [1] and Kuo-Chang Lu [1,2,*]

1 Department of Materials Science and Engineering, National Cheng Kung University, Tainan 701, Taiwan; jinnii10377@yahoo.com.tw (Y.-T.K.); young263263@gmail.com (S.-M.Y.)
2 Center for Micro/Nano Science and Technology, National Cheng Kung University, Tainan 701, Taiwan
* Correspondence: gkclu@mail.ncku.edu.tw; Tel: +886-6-275-7575 (ext. 62920)

S1 BET surface area

Figure S1. shows the Nitrogen adsorption-desorption isotherms and pore size distribution. Also, Table 1 reveals the three parameters in the measurements by BET and BJH.

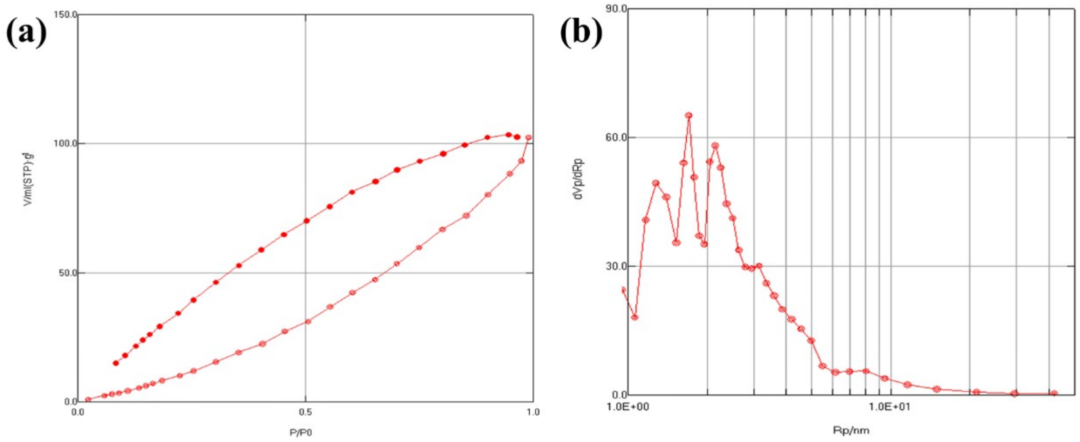

Figure S1. (a) Nitrogen adsorption-desorption isotherms and (b) corresponding pore size distribution curve of the CuO-CuS nanowires.

Table 1. BET surface area and pore size of the CuO-CuS nanowires.

Samples	Surface Area (m^2/g)	Pore Size (nm)	Pore Volume (cm^3/g)
CuO-CuS	135.24	1.7	186.23

S2. Photocatalytic decomposition of methylene blue by CuO-CuS nanowires.

The CuO-CuS nanowires decomposed the MB under visible light four times as shown in Figure S2.

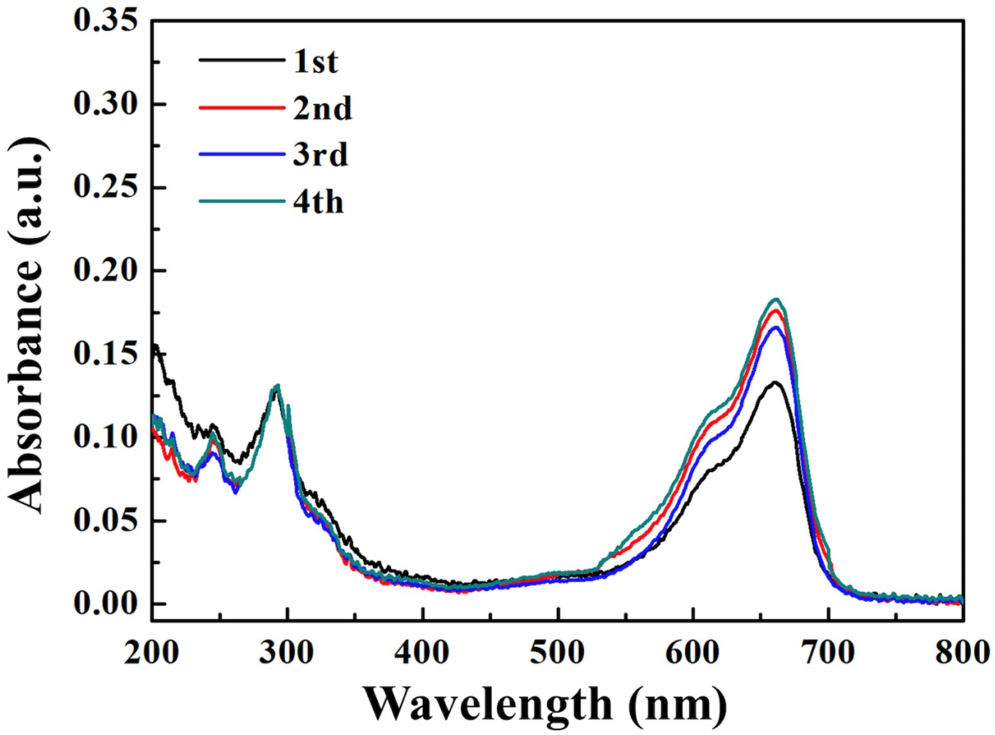

Figure S2. UV-Vis absorbance spectra for cycling degradation of MB aqueous solutions by CuO-CuS nanowires.
